# Anaerobic Metazoans: No longer an oxymoron

**DOI:** 10.1186/1741-7007-8-31

**Published:** 2010-04-06

**Authors:** Lisa A Levin

**Affiliations:** 1 Integrative Oceanography Division, Scripps Institution of Oceanography, La Jolla, CA 92093-0218, USA

## Abstract

The sediments of a deep-sea hypersaline and sulfidic Mediterranean basin have yielded an unexpected discovery, the first multicellular animals living entirely without oxygen. Reported by Danovaro *et al.* in BMC Biology, these three new species of Loricifera add a new and remarkable dimension to anoxic ecosystems previously thought to support only unicellular life.

## 

Few environments on earth lack life in some form; microbes appear to be ubiquitous, with a presence from the atmosphere [1] to the deep subsurface ocean [2]. But extreme settings often lack multicelluar organisms. Some of the most extreme environments on earth can be found in the deep ocean. Excessively high salinities in brine pools, toxic sulfide levels within methane seep sediments, high metal concentrations in hydrothermal vent fluids, high pressures at the bottom of trenches, and anoxic sediments in isolated basins and midwater oxygen minimum zones are examples. Prokaryotic organisms (without nuclei) are known to inhabit all of these settings, and single-cell eukaryotic organisms (protozoa), most noticeably ciliates and foraminifera, are also recorded in most extreme deep-sea environments. Anoxic and dysoxic environments in particular host a reduced diversity of protozoans [3]. These protozoans have a range of adaptations that often involve symbioses [4] and the ability to store and respire nitrate, although most taxa that do this appear to be facultative anaerobes [5]. To date, however, no one has found metazoans capable of living and reproducing entirely in the absence of oxygen. This has changed with the discovery by Danovaro *et al.*[6] of viable loriciferans in a hypersaline, anoxic basin of the Mediterranean Sea.

Loricifera are small (< 1 mm), exclusively marine meiofauna that belong to a relatively recently described marine phylum. Although there are only 22 described species, they have been recorded from a broad range of depths and settings ranging from shallow, coastal waters to methane seeps and hydrothermal vents, to the Izu-Ogasawara trench off Japan [7,8]. Loriciferans are an unlikely candidate for the honor of being the first anoxy-philic metazoan. Low-oxygen sediments have been studied extensively and loriciferans are rarely reported [9]. Whether they were overlooked or are exceedingly rare and thus not sampled is unclear. Perhaps scientists have been looking for them in all the wrong places. In the L'Atalante Basin the loriciferans were sampled with other metazoans (copepods and nematodes) but Danovaro *et al.*[6], using a protein binding stain, fluorogenic probes, and radiolabel uptake experiments, determined that only the loriciferans were alive and metabolically active at the time of collection. They found not one, but three new species of Loricifera, living at the highest densities ever reported for this group. Some of the individuals contained a single large oocyte, and empty moults were also present, providing strong evidence that these populations are reproducing in place under anoxic conditions.

Why is this finding important? These metazoans have had to cope with multiple physiological stresses - extreme salinity, toxic sulfide levels and the absence of oxygen. Evolving adaptations to any one of these would be a challenge. Clues to the success of the Loricifera found by Danovaro *et al.*[6] may lie in part with the ultrastructure of their cells. Their mitochondria have been replaced with hydrogenosome-like structures, organelles that apparently evolved from mitochondria and are only previously known from protozoans inhabiting anaerobic environments. The additional presence of rod-shaped structures in close proximity to the hydrogenosome-like organelles raises questions about microbial symbioses, perhaps evolved from associations with Archaea. That Archaea might play a role in adaptation to anoxia would not be surprising. They are the masters at harnessing energy in anoxic environments [10] and apparently act in association with hydrogenosomes inside ciliates [4], which themselves may be symbionts [11]. If such an association occurred directly in the cells of marine metazoans, without a protozoan intermediary, this would be very exciting indeed!

The findings by Danovaro *et al.*[6] may at first glance seem to be idiosyncratic, relevant only to an unusual set of environmental conditions. But there are major implications here that extend beyond the specifics of this study. Perhaps most apparent is that the deep sea remains full of novel ecological settings which have yet to be studied, and in some cases they have yet to be discovered. When they are found, and scientists look carefully, they nearly always find previously undescribed eukaryotic species with novel adaptations and metabolic pathways. Often these form completely new assemblages or ecosystems. Chemosynthetic communities at hydrothermal vents and methane seeps are outstanding examples [12]. The three loriciferan species documented by Danovaro *et al.*[6], representing three genera, form another unique assemblage.

What does it mean to have metazoans inhabiting anoxic environments? As exploration of low-oxygen settings has intensified, it has become apparent that metazoan life can persist at very low oxygen levels of only a few micromolar per kg. In such settings metazoans can play important functional roles in the recycling, bioturbation and burial of carbon and nitrogen [13,14,5]. Often the key to their success appears to be association with epibiotic or endosymbiotic bacteria [13,15]. But the symbiotic bacteria are oxidizers that require at least some oxygen. With the discoveries of Danovaro *et al.*[6] it appears that metazoans can live where oxygen is absent. The L'Atalante Basin loriciferans join a select group of foraminiferans and ciliates in achieving an obligate, anaerobic existence.

Are there more metazoan taxa out there that can live without oxygen? Almost certainly. The finding by Danovaro *et al.*[6] offers the tantalizing promise of metazoan life in other anoxic settings, for example in the subsurface ocean beneath hydrothermal vents or subduction zones [2] or in other anoxic basins. Good places to look might be the Cariaco Basin and the Black Sea, as well as the many borderland basins off southern California and Baja California. The finding of several new rotifer species, taxa rare in open water or deep marine settings, in deep sulfidic sediment layers at methane seeps [16] points to another meiofaunal group with possible adaptations to anaerobic existence.

Are there metazoans on other planets with atmospheres different from our own? Our ability to answer this question would be strengthened considerably by more intensive studies of animal - microbe interactions in extreme settings of our own inner space - the deep ocean.
